# Older Adults Show Reduced Spatial Precision but Preserved Strategy-Use During Spatial Navigation Involving Body-Based Cues

**DOI:** 10.3389/fnagi.2021.640188

**Published:** 2021-04-12

**Authors:** Andrew S. McAvan, Yu Karen Du, Alexis Oyao, Stephanie Doner, Matthew D. Grilli, Arne Ekstrom

**Affiliations:** ^1^Human Spatial Cognition Laboratory, Psychology Department, University of Arizona, Tucson, AZ, United States; ^2^Evelyn McKnight Brain Institute, University of Arizona, Tucson, AZ, United States

**Keywords:** spatial precision, spatial navigation, aging, impairment, allocentric, egocentric, virtual reality

## Abstract

Older adults typically perform worse on spatial navigation tasks, although whether this is due to degradation of memory or an impairment in using specific strategies has yet to be determined. An issue with some past studies is that older adults are tested on desktop-based virtual reality: a technology many report lacking familiarity with. Even when controlling for familiarity, these paradigms reduce the information-rich, three-dimensional experience of navigating to a simple two-dimensional task that utilizes a mouse and keyboard (or joystick) as means for ambulation. Here, we utilize a wireless head-mounted display and free ambulation to create a fully immersive virtual Morris water maze in which we compare the navigation of older and younger adults. Older and younger adults learned the locations of hidden targets from same and different start points. Across different conditions tested, older adults remembered target locations less precisely compared to younger adults. Importantly, however, they performed comparably from the same viewpoint as a switched viewpoint, suggesting that they could generalize their memory for the location of a hidden target given a new point of view. When we implicitly moved one of the distal cues to determine whether older adults used an allocentric (multiple landmarks) or beaconing (single landmark) strategy to remember the hidden target, both older and younger adults showed comparable degrees of reliance on allocentric and beacon cues. These findings support the hypothesis that while older adults have less precise spatial memories, they maintain the ability to utilize various strategies when navigating.

## Introduction

Numerous studies indicate that older adults show reduced performance on spatial memory tasks compared to younger adults (Kirasic, 1991; Newman and Kaszniak, 2000; Moffat et al., 2001; Moffat and Resnick, 2002; Head and Isom, 2010; Rodgers et al., 2012; Wiener et al., 2013; Allison and Head, 2017; Zhong et al., 2017). An important question, however, regards the nature of these spatial deficits. While control analyses in several of the studies mentioned above suggest that the deficits are unlikely to be due to perceptual or motor issues alone, whether such impairments relate to reductions in the fidelity of spatial memory representations or impaired strategy use remains unclear. For example, it could be that older adults are just as capable as younger adults at employing a strategy of referencing to external landmarks (often termed allocentric navigation), but that their memories are simply less detailed and precise (see Koen and Yonelinas, 2014; Koen and Rugg, 2019; Korkki et al., 2020). Additionally, given that older adults may have less computer and virtual reality exposure than younger adults (e.g., Head and Isom, 2010), and that some real-world studies have suggested largely conserved navigation abilities in older adults (Kirasic, 1991), it is important to test older adults with a fuller range of body-based cues than desktop VR permits.

Allocentric navigation refers to finding a target using multiple landmarks, often ones that are outside of the boundaries of the experiment and placed at a distance (termed “distal” cues). In support of the idea that older adults show impairments in certain navigational search strategies, some studies that looked at how effectively older adults use distal cues to remember a location suggest a specific deficit in allocentric navigation (Moffat and Resnick, 2002; Antonova et al., 2009; Rodgers et al., 2012; Zhong et al., 2017). In contrast, some of these same studies show intact memory for hidden targets when strategies involve a proximal cue indicating its location. Similar findings have also been shown in older rats navigating the Morris water maze: older rats showed impaired navigation using distal cues, but largely intact spatial memory using proximal cues (Barnes et al., 1980; Gallagher et al., 1993, 2015). Together, these findings suggest that aging may selectively impact the ability to employ an allocentric strategy involving the use of distal cues to remember a hidden target (Moffat, 2009; Lester et al., 2017).

Notably, however, many of these studies in humans did not control for differences in allocentric vs. egocentric navigation. Specifically, when testing in the virtual Morris Water Maze, the use of proximal cues as demonstrated in these past studies is less likely to provide insight into egocentric representations of space and is more likely to measure a form of beaconing (response learning). This is because beaconing involves moving to a proximal cue without the need to remember any specific (egocentric) turns. Even in situations involving environments other than the Morris water maze, taking a right or left turn in response to a cue (e.g., Rodgers et al., 2012) is unlikely to evoke an egocentric representation because it is a response to a cue rather than a specific memory for self-referenced coordinates (Ekstrom and Isham, 2017). In addition, several other studies have challenged the idea of a strict dichotomy between allocentric and egocentric navigation and how this is typically examined in navigation tasks (Wolbers and Wiener, 2014; Zhong and Kozhevnikov, 2016; Ekstrom and Isham, 2017; Ekstrom et al., 2017; Starrett and Ekstrom, 2018). Therefore, to compare performance on memory representation vs. strategy, it is important to test memory for a hidden target from a repeated vs. a novel viewpoint (Eichenbaum et al., 1990; Wolbers and Wiener, 2014; Kolarik et al., 2018). Here, we test older adults in situations that more directly interrogate one navigational strategy compared to another by testing memory for a target location from a specific viewpoint (more egocentric) vs. a novel viewpoint (more allocentric).

Another concern related to understanding egocentric and allocentric navigation in older adults is that the majority of studies that have demonstrated impairments in older adult allocentric navigation have involved tasks rendered on a desktop computer. An issue with testing older adults in virtual reality rendered on a desktop computer is that they may have less experience with such interfaces in the first place, for example, a few studies showed lower familiarity ratings with desktop computers and VR usage in older compared to younger adults (Head and Isom, 2010; Rodgers et al., 2012). Desktop VR interfaces also do not capture the full navigation experience because rather than including walking and turning that is afforded by wireless immersive VR (and real-world navigation), desktop VR requires users to be stationary and control an on screen avatar (from either first or third person). Even in cases in which extensive preexposure to a computer is provided to participants in an attempt to match age groups (Moffat and Resnick, 2002), cohort effects (differences in the age at which one group first learned about computers) are likely to exert an influence on how well older adults interface with desktop VR. In addition to the lack of ambulatory cues, desktop VR involves learning about 3-D spatial environments on a 2-D monitor. Previous studies have also suggested that younger adults show differences in their levels of spatial knowledge acquisition in real-world environments and situations involving wider access to body-based cues when compared to virtual environments and situations with fewer body-based cues (Chance et al., 1998; Klatzky et al., 1998; Richardson et al., 1999; Hejtmanek et al., 2020), although some of these differences may be negligible for well-learned environments (Huffman and Ekstrom, 2019). Given that older adults may struggle with computers, particularly VR, and even young adults show differences in learning based on whether VR is rendered on head-mounted displays with body-based cues or desktop VR, it is important to test older adults with VR interfaces that more fully mimic and approximate their previous real-world experiences.

In support of the importance of how older adults learn about a spatial environment, several studies conducted in real-world environments have shown somewhat different findings from those conducted with 2-D VR on a computer. In one seminal study conducted by Kirasic (1991), older and younger adults searched for objects in a new and familiar supermarket. While older adults showed less accurate distance estimations and route efficiency for objects in the new supermarket compared to younger adults, their distance estimations were highly correlated with actual distances in the familiar supermarket and matched those of younger adults. Their routes in the familiar supermarket were also of comparable efficiency to younger adults. Older adults also show similar performance as younger adults in spatial memory when given rest periods (Craig et al., 2016) and in discriminating critical landmarks for navigation (Zhong and Moffat, 2016). Thus, at least under some testing situations, older and younger adults show largely comparable navigational abilities, particularly under situations in which they have sufficient exposure.

In this study, we tested older and younger adults in an approximately 6 × 6 m room in which participants viewed an environment while wearing a wireless head-mounted display. The environment, like the virtual Morris water maze, consisted of distal cues (mountains) and target objects that participants learned the locations of during acquisition trials, and were then tested on during probe trials. The study occurred within a single session and took less than 3 h to complete. Based on numerous studies demonstrating that older adults show impairments in spatial navigation, particularly in new environments, we predicted that older adults would show impairments in their memory for learned target locations regardless of whether navigation was egocentric (same viewpoint as trained) or allocentric (new viewpoint). At the same time, because participants navigated using their bodies, distal cues were prominent and easily identifiable on the head-mounted display. In addition, we disoriented participants on every trial by leading them around on a random path through the environment while the display rendered by the HMD was blacked out. This served as a control sometimes employed in rodent studies of the Morris water maze (Dudchenko et al., 1997) to ensure that participants did not maintain their bearing from trial-to-trial as a means to remember the hidden target. We compared navigation from repeated and novel start points to test whether older adults would show significantly greater spatial memory deficits for putative egocentric vs. allocentric navigation. As an additional measure, we included a condition in which one of the four distal mountain cues moved. If older adults differentially rely on response strategies, then older adults should show a strong tendency compared to younger adults to follow a single distal cue rather than the three that do not move.

## Materials and Methods

### Participants

We recruited 15 (11 female) undergraduate students from the University of Arizona Psychology program whose ages ranged from 18 to 28 years old with a mean age of 19.80, and 18 (nine female) older adults from the surrounding Tucson area whose ages ranged from 66 to 82 years old with a mean age of 74.05. Due to technical issues (insufficient battery charge on the wireless head-mounted display), three younger and three older participants had their data excluded. Data from 12 younger adults whose ages ranged from 18 to 28 years old with a mean age of 20 and 15 older adults whose ages ranged from 66 to 82 years old with a mean age of 74.27 were used in subsequent analyses. The younger adults received class credit for participation in the study while the older adults received monetary compensation. All participants had normal or corrected-to-normal color vision, normal or corrected-to-normal hearing, and reported no history of cardio-vascular problems or motion sickness. Written informed consent was obtained before the experiment, and the methods were approved by the Institutional Review Board (IRB) at the University of Arizona (1807727476A014).

Older adults were characterized as cognitively normal according to a neuropsychological profile approach that has been shown to improve diagnostic accuracy for mild cognitive impairment relative to traditional cognitive screening or single test score approaches (Bondi et al., 2014). Consistent with past work (Bondi et al., 2014; Grilli et al., 2018), two neuropsychological test scores were selected from multiple cognitive domains, namely learning and memory (Rey–Osterrieth Complex Figure Test long delay recall, Rey, 1941; California Verbal Learning Test Second Edition long delay free recall, Delis et al., 2000), attention/processing and speed/executive functioning (Trail Making Test Part A and B total time, Reitan and Wolfson, 1993), language (Animal fluency total correct from the Controlled Oral Word Association Test, Benton, 1969; Boston Naming Test total correct, Goodglass et al., 2001), and visuospatial functioning (Rey-Osterrieth Complex Figure Test copy score, Rey, 1941; Block Design from the Wechsler Adult Intelligence Scale Fourth Edition, Wechsler, 2008). Individuals were considered cognitively normal if neither of the following were met: (1) they performed more than one standard deviation below the age-corrected (and education-corrected if available) normative mean on both scores in one cognitive domain, or (2) they performed more than 1 standard deviation below the age-corrected (and education-corrected if available) normative mean on one test in three cognitive domains (Table 1). As shown in Table 1, our group of older adults, as seen from their z-scores and respective standard deviations, which were based on comparing them to age- and education-matched (if available) normative samples, were within the normal range of intellectual and cognitive function as they had mean z-scores ranging from −0.65 to 1.33 across our battery.

**TABLE 1:** Mean (standard deviation in parentheses) Z-score performance on the neuropsychological tests for older adults.

	Mean (SD)
**Learning and memory**	
CVLT-II LDFR	0.56 (1.01)
RCFT LDFR	−0.49 (1.12)
**Attention/executive functioning**	
Trails A	−0.02 (1.03)
Trails B	−0.08 (0.80)
Language	
BNT	1.33 (1.02)
Animals	0.06 (1.10)
**Visuospatial functioning**	
WAIS-IV Block Design	0.73 (0.91)
RCFT Copy	−0.65 (0.78)

*SD, standard deviation; CVLT, California Verbal Learning Test; RCFT, Rey Complex Figure Test; BNT, Boston Naming Test; LDFR, long delay free recall; WAIS-IV, Weschler Adult Intelligence Scale. A *z*-score outside the range of ±1.7 would indicate being cognitively impaired in the measure while a z-score within this range indicates being cognitively normal in the measure.*

### Materials

**FIGURE 1:**
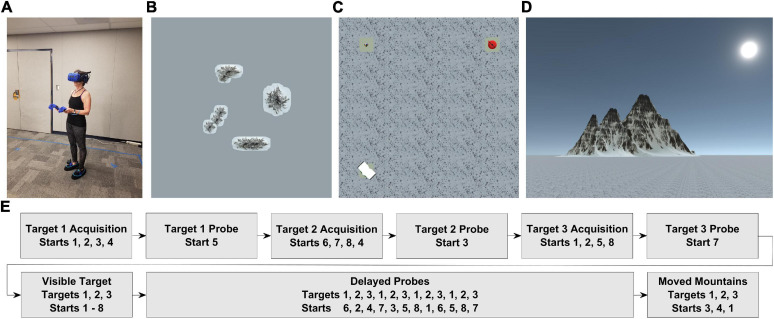
Overview of task setup **(A)**. Older adult fitted with wireless HMD, battery pack, two controllers, and two foot covers with trackers **(B)**. Bird’s-eye-view of entire virtual environment, including the 4 distal mountains (entire environment approximately 750 × 750 m in size) **(C)**. Bird’s-eye-view of navigable virtual environment (approximately 5 × 5 m in size), with all three targets visible **(D)**. Point-of-view from the participant within the virtual environment **(E)**. Trial list with corresponding targets and starting locations.

The virtual environment (Figures 1A–D) and experimental tasks (Figure 1E) were built in Unity 3D (Unity Technologies ApS, San Francisco, CA) using the Landmarks virtual reality navigation package (Starrett et al., 2020). The navigable virtual environment was approximately 5 × 5 m in size, with the full space spanning 750 × 750 m. Four distally rendered mountains (unevenly spaced) were visible from within the 5 × 5 m space. In addition, the environment contained a snow-covered floor and three unique objects (book, puzzle cube, and teapot) on pedestals which served as the hidden targets for navigation (see Procedures).

To simulate the immersive experience of being in a mountainous environment, we used the HTC Vive Pro head-mounted display (HMD) in conjunction with the HTC Wireless Adapter (HTC, New Taipei City, Taiwan) to allow for untethered, free ambulation. The Vive Pro displayed stimuli at a resolution of 1,140 × 1,600 pixels per eye, 90 Hz refresh rate, and a 110° field of view, while the Wireless Adapter delivered data over a 60 GHz radio frequency for up to 7 m. To record responses from participants and allow interaction with the virtual environment, we used the two handheld HTC Vive controllers (HTC, New Taipei City, Taiwan). We also used two HTC Vive trackers (HTC, New Taipei City, Taiwan) to allow for rendering of virtual shoes and foot tracking. The tasks were run on a custom-built computer with an NVIDIA GeForce Titan Xp graphics card (NVIDIA Corp., Santa Clara, CA, United States).

### Procedures

After reading and signing the consent form, participants were blindfolded and led into the navigation space. The purpose of the blindfold was to prevent participants from seeing the size and shape of the room they would be walking in. Then, they were fitted with the wireless HMD, two handheld controllers, a clip-on battery pack that powered the wireless HMD, and two trackers attached to their shoes. Participants were then immersed in a practice virtual environment similar to the main task described here. The practice portion required participants to freely navigate around a small circular room for five trials before being prompted to find and remember the location of a single target object for another five trials. The practice session lasted approximately 10 min.

After the practice, participants were tasked with completing five blocks of a navigation task (Figure 1E) with breaks offered in-between. Our study design was based on an earlier study using the virtual Morris water maze with amnesia patients conducted with desktop VR (Kolarik et al., 2018). In each task block, participants were verbally and visually instructed on what to do in the task and provided with reminders if they requested them. Throughout the experiment, the participants heard white noise on the headphones attached to the HMD to prevent sound cues from providing location or orientation information. After completion of each trial, participants were briefly disoriented by guiding them around the environment without vision. This ensured that participants could not track their bearing and movements through the environment, therefore requiring them to use their memory for the locations rather than simply recapitulating their learned responses based on being oriented. The resulting design was kept constant between all subjects resulting in a repeated measures design with six conditions: three for each start type (same start, different start, moved mountain start) between each age group (YA, OA) across each navigation block (blocks one through five). All three of the targets had equal exposure across participants, and the order of the starting locations was kept constant across participants.

### Navigation Task: Acquisition Blocks

Participants were familiarized with each of the three targets by performing 16 trials of acquisition learning across four different start locations. Before each trial, the participant was disoriented by being randomly led around the navigation space for 30 s while the virtual environment (VE) was blacked out. At the end of the disorientation procedure, the participant was then placed at one of eight start locations (Figure 1E). Each trial began with text indicating the navigation goal (e.g., “Please find the book”). Participants then freely navigated the environment until the target object appeared after 30 s. After the first trial of each acquisition block, the participants were given the option to make the target appear before the 30 s if they felt like they knew where the target was. That is, if the participant was confident about the location of the target, they could press a button on their controller, thus making the target object appear. Pressing the button would record their location and time of press as a response. Once the target appeared, the participants would then walk up to the target and interact with it by using their controller to make contact. This procedure was kept constant for the first 16 trials of each acquisition block, after which they would experience a single probe trial, followed by an optional break to help mitigate any fatigue or motion sickness.

#### Navigation Task: Single Probe Trial at End of Each Acquisition Block

After the first 16 trials of each acquisition block, participants then performed a single probe trial. In these probe trials, participants were placed at a new start location for the block, and then walked to where they thought the prompted target was, pressing a button on the controller to make the target appear (mirroring the probe trials within the delayed probe block). The purpose of these probe trials was to test their ability to change to a new start location immediately after experiencing the same target from numerous repeats of the same start location.

### Navigation Task: Visible Target Block

After the three acquisition blocks, participants then completed one block of eight visible target trials. These trials served as a control for motivational and potential sensorimotor deficits in performing the task. Because the target was continuously visible for the entire trial, participants could use a simple beaconing strategy to locate the target. The visible trials tasked participants with finding each target in sequential order while at the same time being positioned at each start location in sequential order (e.g., target 1 and start 1, target 2 and start 2, target 3 and start 3, …, target 2 and start 8). Before each trial, the participant was again disoriented for 30 s while the virtual environment was blacked out and each trial began with text indicating the navigation goal. The target they were tasked with finding was visible from the start of each trial, and they simply had to walk to it and interact with it to complete a trial. After the visible target block, participants were offered another break to help mitigate any fatigue or motion sickness.

### Navigation Task: Delayed Probe Block

Following the visible target block, participants performed 15 trials that tested their memory of the targets they previously learned. In the delayed probe trials, participants found each target in sequential order while starting from a pre-determined randomized start location. Some of these start locations were repeated from acquisition while some others were novel start locations. There were two trials in which participants received a target-viewpoint pairing that was exactly the same as one previously seen in the acquisition phase. There were four trials in which participants received a target-viewpoint paring that was never seen before. Before each trial, the participant was disoriented and then prompted with text indicating the navigation goal. The target they were tasked with finding did not appear until they navigated to its remembered location and pressed a button on their controller to mark their response; pressing the button would record their location and time of press. After pressing the button, the actual location of the target would appear.

#### Navigation Task: Moved Mountains Probe Trials

Immediately following the delayed probe trials were three probe trials with the same design as those previously experienced, with the only difference being that one of the distal mountains was randomly rotated 20°clockwise or counter-clockwise around the target. The purpose of rotating one of the distal mountains was to explore how manipulation of a subset of distal navigational cues affects accuracy. If participants used a single mountain as a beaconing cue, they would be strongly influenced by its new position. In contrast, if participants used a combination of the mountains to derive allocentric coordinates, they would show greater reliance on the three unmoved mountains.

### Data Collection

Throughout the entire experiment, we collected the position and rotation data of the HMD in the environment at a sampling rate of approximately 10 Hz. We also sampled the position and rotation data of trackers attached to each of the participants feet at a sample rate of approximately 10 Hz. Due to issues with battery life and trackers rotating during walking, we excluded the foot tracking data from our analysis.

### Data Analyses/Statistics

Data processing and analyses were completed in Excel 2019 (Microsoft Corportation, Redmond, WA, United States), MATLAB 2020a (MATLAB, 2020), and RStudio (R Core Team, 2020; RStudio Team, 2020). Individual paths for younger adults and older adults were calculated by summing the distances between each sampled data point and split into various measures including the path involving the start location to the response location, the path of the response location to the target location, and total path length. Distance to target (or “distance error”) was the shortest path between the participant’s response and the target (bird-flight distance or Euclidean distance). To understand some of these trends in the raw data in more depth, we compared younger and older adult distance errors using a pooled analysis with group statistics. To determine if participants weighted the moved mountain over the other three static mountains, we looked at their response distance from where the target would be if it moved with the mountain (EMM) over that same distance added to their response distance from the actual static location of the target (EMM + E3M). A value of 1 would indicate completely weighting the three unmoved mountains over the single moved mountain. A value of 0 would indicate completely weighting the single moved mountain over the unmoved three mountains. Values in-between indicate partial weighting of both. A value of less than 0.50 would indicate that participants weighted the moved mountain more than the static mountains, a value of greater than 0.50 would indicate that participants weighted the static mountains more than the moved mountain, and a value of 0.50 would indicate equal weighting. To determine the strength of our effects, we included Bayes statistics (Rouder et al., 2009). Specifically, we calculated Bayes Factors using the BayesFactor package in R with default parameters (Morey and Rouder, 2018). We used a Bayes factor *BF*_10_ to indicate favourability for the alternative hypothesis, and a Bayes Factor *BF*_01_ to indicate favorability for the null hypothesis. Regardless of the hypothesis, the larger the Bayes Factor the greater the support. For our purposes, a Bayes factor of 1–3 is considered anecdotal support, 3–10 is considered substantial support, and > 10 is considered strong support (Jeffreys, 1961).

## Results

### Older Adults Remember the Target Location Less Precisely During Acquisition Although Switch Viewpoints Comparably to Younger Adults

**FIGURE 2:**
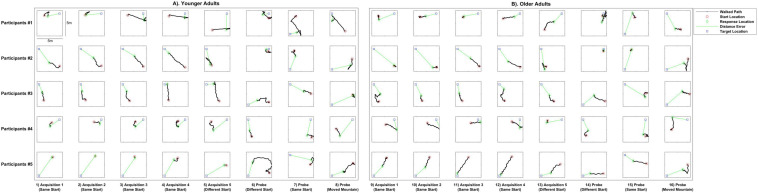
Example paths walked by younger and older adults on individual trials. Columns 1–8 show younger adult paths, Columns 9–16 show older adult paths. Columns 1–4 and 9–12 show four trials of acquisition from the same starting point, columns 5 and 13 show an acquisition trial from a novel view point, and columns 6–8 and 14–16 show walked paths on three different types of probe trials: a new start location not seen during acquisition, a start location that was previously seen during acquisition, and one involving movement of a distal mountain. The red circles show where the participant started, the black lines show the path the participant walked, the green diamonds show where the participant made their response, and the blue squares show the target location. Green lines indicate the Euclidean distance from the response to target location, which correspond to the distance error shown in Figures 3–5.

We first investigated the paths taken by both older and younger adults on specific trials to better understand any differences as a function of start point and age (Figure 2). As can be seen in almost all trials comparing young (left panels) with older adult (right panels) trajectories, and particularly during learning (acquisition trials, columns 1–5 and columns 9–13), older adults placed the target at a greater distance from its actual location than younger adults (green line indicates Euclidean distance between the response and the target).

**FIGURE 3:**
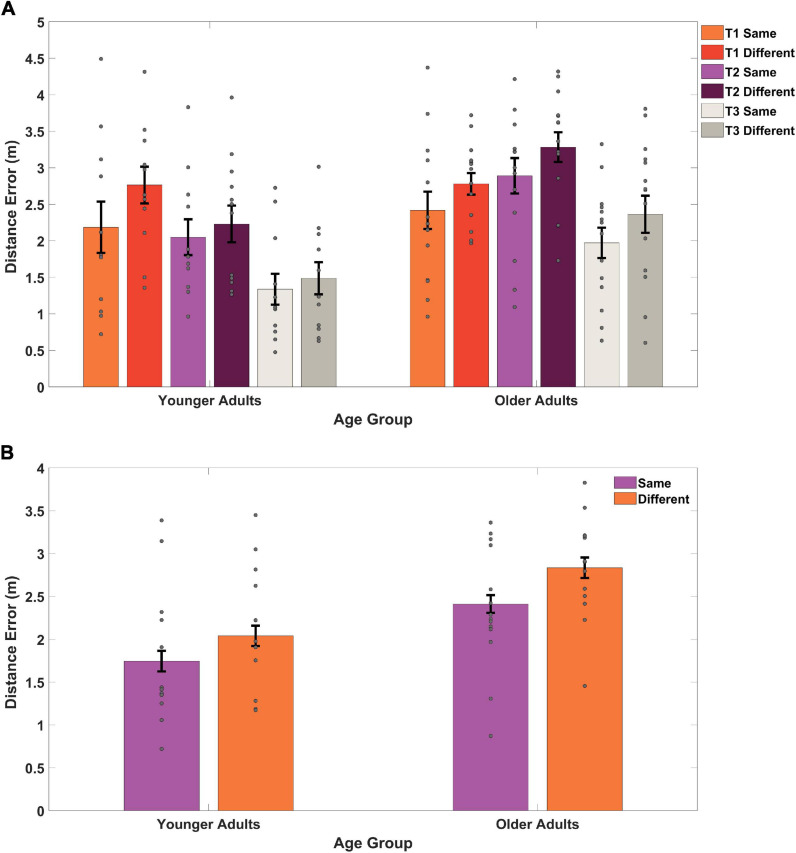
Distance between remembered location and actual location, or distance error, on acquisition trials **(A)** for each of the three different targets across two different start types **(B)**, and collapsed across targets for start type. “Same” refers to a repeated viewpoint and “Different” refers to a novel viewpoint. T1 refers to target 1, T2 refers to target 2, and T3 refers to target 3. Gray dots represent individual subject data, bars indicate the mean, and error bars represent the standard error. As a group, older adults remembered the target location as further from its actual location than younger adults.

Two trends were evident in the data: older adult placement of the target location was further from the actual object location than younger adults both when examining across targets, and when targets were collapsed (Figures 3A,B). This analysis also revealed overall lower accuracy during acquisition when learning the target location from a different start location in both groups (Figure 3A), both when considering each target separately (Figure 3A) and when averaging over targets (Figure 3B). To understand these effects during acquisition statistically, we performed a mixed-effects ANOVA, with age group as a between-subject variable, start location as a within-subject variable, and response distance from the target as the dependent variable. We found a significant main effect of age group [*F*(1,24) = 7.72, *p* = 0.010, η*_p_^2^* = 0.243, *BF_10_* = 24.50], demonstrating that younger adults placed the target closer to its actual location than older adults. This suggests that older adults were less precise in terms of their remembered distance of the target compared to its actual location (*M* = 2.61, *SD* = 0.69) than younger adults (*M* = 1.90, *SD* = 0.77), consistent with our observations from the path data in Figure 2.

We also found a significant main effect of start location [*F*(1,24) = 8.05, *p* = 0.009, η*_p_^2^* = 0.25, *BF_01_* = 1.09] in which participants performed better on the same starting location trials (*M* = 2.10, *SD* = 0.80) compared to the different starting location trials (*M* = 2.47, *SD* = 0.77). We did not, however, find an interaction effect between age group and start location [*F*(1,24) = 0.99, *p* = 0.330, η*_p_^2^* = 0.04, *BF_01_* = 2.29]. Together, these findings suggest that older adult memory for the hidden targets was less precise than younger adults during acquisition, consistent with our primary prediction. We also found that, at least initially during acquisition, all participants performed worse on the same vs. a different start point. We did not, however, find an interaction effect, suggesting that age did not impact the ability to remember the target from a new start location, consistent with the idea that aging should not disproportionately affect strategies involved in allocentric navigation.

### Older Adults Remember the Target Location Less Precisely During Delayed Probe Trials but Learn to Effectively Generalize Their Memory of the Target to Novel Start Locations

**FIGURE 4:**
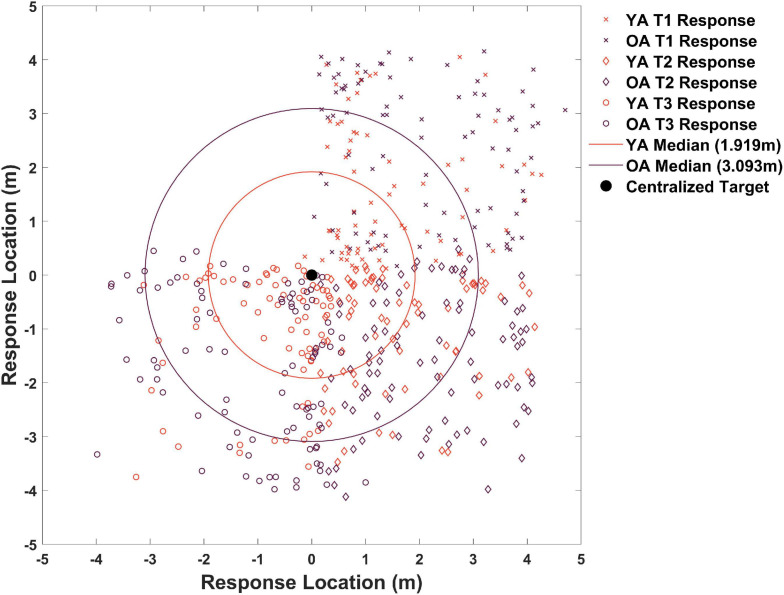
Memory for target locations across all trials. Responses in orange correspond to younger adults and responses in plum correspond to older adults. X’s are all probe responses for target 1, diamonds are all probe responses for target 2, and circles are all probe responses for target 3. All responses are centralized around (0,0) to better show distance from a single centralized target. Older adults remembered the target as further from the actual location than younger adults. Note: the upper left quadrant has few data points because we employed three targets.

We then considered delayed probe trials, which occurred after all acquisition blocks plus a delay. We again found a tendency for older adults to place the target less precisely than younger adults (Figure 2 compare right vs. left panels, columns 6–8). To better understand any groups differences in precision during probe trials, we plotted all participant responses for where they remembered the target was on each of the 18 total probe trials. As shown in Figure 4, half of all younger adult responses (red dots) fell within 1.92 m of the centralized target and half of all older adult responses (blue dots) fell within 3.09 m of the centralized target. Due to a positively skewed distribution within each group’s responses, we plotted the median rather than the mean (YAs *M* = 2.06, OAs *M* = 2.93). We then compared responses in terms of Euclidean distance to the target between same and different starts across age groups to determine what influence –if any—these different starting conditions have on participant accuracy.

We then performed two mixed-effects ANOVAs with age group as a between-subject variable, start location (same and different) as the within-subject variable, and response distance from the target as the dependent variable. We looked at both same and different start locations. Same probe trials involved start locations that were learned during acquisition (but not tested on acquisition probe trials), while different probe trials involved a completely new start point that had not yet been experienced. Due to only having two same trials (65 and 70), we matched those with two of the four different trials (66 and 67).

**FIGURE 5:**
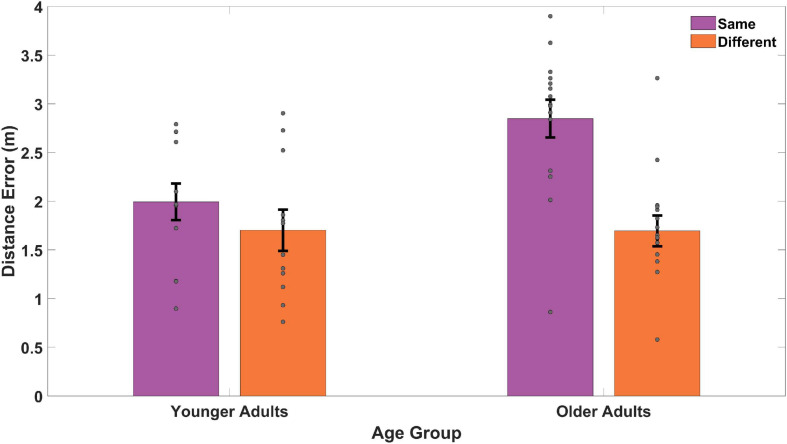
Memory for target location compared to the actual location for same and different start points during probe trials. The two same trials (65 and 70) were matched with two different trials (66 and 67) for all younger adults and older adults. Gray dots represent individual subject data, bars indicate the mean distance error, and error bars represent the standard error. YAs performed better than OAs with both groups doing better on different (or novel) viewpoints than same (or repeated) viewpoints.

Same and different start point search accuracy is shown in Figure 5 for younger and older adults. As is evidenced in Figure 5, during probe trials, both groups showed numerical trends to perform slightly worse on same vs. different start points, likely an effect of repeated exposure to multiple start points. A mixed-effects ANOVA revealed a significant main effect of age group [*F*(1,25) = 12.65, *p* = 0.002, η*_p_^2^* = 0.34, *BF_10_* = 674.22], where younger adults again performed better overall than older adults. This suggests that older adults were again less precise (*M* = 2.81, *SD* = 0.60) in their memory for the position of the hidden target than younger adults (*M* = 2.01, *SD* = 0.68) regardless of start type. We did not find a significant main effect for start type [*F*(1,25) = 0.05, *p* = 0.821, η*_p_^2^* = 0.002, *BF_01_* = 3.63] nor did we find a significant interaction effect between age group and start location [*F*(1,25) = 0.20, *p* = 0.658, η*_p_^2^* = 0.008, *BF_01_* = 2.87]. These findings suggest that with sufficient exposure to different start points, both younger (Same *M* = 1.99, *SD* = 0.92; Different *M* = 1.70, *SD* = 1.04) and older adults (Same *M* = 2.85, *SD* = 1.06; Different *M* = 1.70, *SD* = 0.85) could generalize comparably well to new start locations.

### Younger and Older Adults Show a Similar Reliance on Multiple Distal Cues and a Single Beacon

**FIGURE 6:**
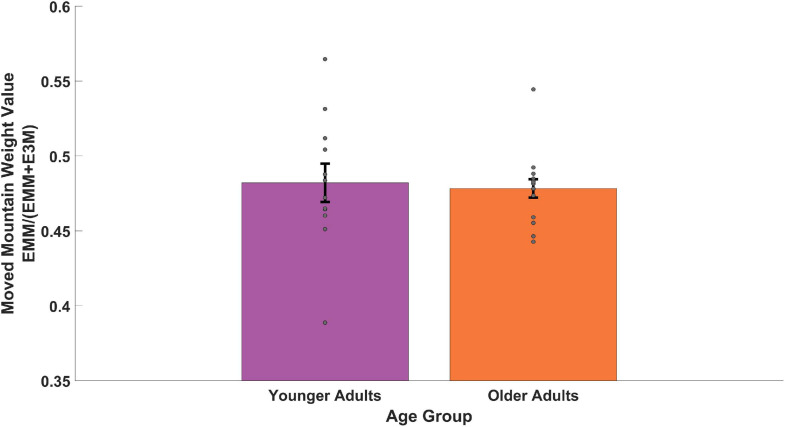
Memory for the target during trials in which one distal cue moved. EMM (see Section 3.5) provides a relative weighting of allocentric vs. beacon cues by assuming the memory for the target moves with the mountain and dividing it by the same distance + the target’s unmoved location. Values less than 0.5 would favor moving with the mountain and values larger than 0.5 would favor utilizing the 3 distal cues.

We then considered the subset of trials in which we explicitly moved one of the distal cues to test the dependence of older and younger adults on combining (“triangulating”) distal cues. To do so, we looked at the relative weighting of a single mountain that moved compared to the three that did not (see Section 3.5 above). As shown in Figure 6, we found that both groups showed a weighted value of less than 0.50 and thus numerically weighted the single moved mountain slightly more than the three static mountains. To compare between the age groups, we ran a Welch’s two-sample *t*-test and found no significant difference between younger and older adults [two-sample *t*(49) = 0.26, *p* = 0.792, *d* = 0.11, *BF*_01_ = 2.71]. These findings suggest that both younger and older participants weighted the moved mountain to comparable extents and that neither group showed a difference in using a response nor an allocentric strategy. To compare if each age group was significantly different from 0.50, we ran a one-sample *t*-test on each. When comparing the younger adults, we found no significant difference between their value and 0.50 [one-sample *t*(11) = 1.40, *p* = 0.189, *d* = 0.40, *BF*_01_ = 1.55]. When comparing the older adults, however, we found a significant difference between their value and 0.50 [one-sample *t*(14) = 3.52, *p* = 0.003, *d* = 0.91, *BF*_10_ = 13.68]. Higher variability in the younger adults (Figure 6, see dots for each subject) makes it difficult to conclude whether they used a strategy involving mixed weighting or one more biased toward the moved mountain, as appeared to be the case for older adults.

Finally, we also compared older and younger adult performance on the visible trials, total movement, and total rotation. We also considered the time taken to find targets and other dependent measures that might differ as a function of age that could confound our interpretations. The visible trials, in particular, were helpful for ruling out any potential perceptual or motor impairments that might accompany age. There were no significant age differences on any of these measures. These findings are shown in Table 2.

**TABLE 2:** No difference in dependent measures related to total walked distance, total rotation, response time, or excess distance (difference of total distance from optimal distance) for delayed probe trials across age groups.

	Young adults	Older adults	*t*-test				Wilcox test	
			
	Mean (SD)	Mean (SD)	T	df	p	d	W	p
Total distance (probe)	6.56 (2.69)	6.02 (2.85)	0.82	24.77	0.42	0.44	119	0.17
Total rotation (probe)	1,698.83 (1,499.75)	1,502.66 (1,086.30)	0.80	18.80	0.43	0.45	99	0.68
Excess distance (probe)	3.24 (2.54)	2.72 (2.63)	0.80	24.82	0.43	0.43	119	0.17
Excess rotation (probe)	1,630.79 (1,501.52)	1,444.30 (1,087)	0.75	19.065	0.46	0.42	99	0.68
Total time (probe)	21,034.50 (10,998.66)	21,238.24 (12,944.43)	0.07	24.99	0.94	0.04	97	0.76
Response Time (Probe)	17,437.43 (9,885.26)	14,632.25 (11,354.74)	0.88	24.98	0.39	0.48	116	0.22
Total distance (visible)	2.88 (1.73)	2.93 (1.86)	0.16	24.94	0.88	0.09	93	0.90
Total rotation (visible)	684.90 (594.23)	767.71 (877.46)	0.56	21.40	0.58	0.30	92	0.94
Excess distance (visible)	0.40 (1.27)	0.57 (1.66)	0.63	24.65	0.53	0.34	89.5	1
Excess rotation (visible)	595.78 (594.51)	678.51 (86,164)	0.57	21.40	0.58	0.30	92	0.94
Total time (visible)	9,532.43 (7,918.10)	11,488.79 (11,667.56)	0.87	21.84	0.39	0.46	78	0.58

*There were also no differences in these measures for visible trials.*

## Discussion

Our study involves several important findings about age-related differences in spatial navigation. Consistent with many studies that compared older and younger adults on spatial memory and navigation tasks (Newman and Kaszniak, 2000; Moffat et al., 2001; Moffat and Resnick, 2002; Head and Isom, 2010; Rodgers et al., 2012; Wiener et al., 2013; Allison and Head, 2017; Zhong et al., 2017; Nilakantan et al., 2018), we found that older adults performed worse overall in terms of the precision of their memories for the hidden targets. Our findings are thus consistent with previous studies in older adults suggesting decrements in spatial memory, particularly during navigation. Our findings therefore support the hypothesis that older adults show impairments in spatial memory relative to younger adults, particularly in the precision of memory for learned locations.

Somewhat in contrast to some previous studies, our findings do not support a selective deficit with age in allocentric navigation. During acquisition trials, both older adults and younger adults showed a comparable decrement in the accuracy of the placed target when tested from a new start location, although older adults showed consistently lower precision in their memory for the targets from both same and new start points. This suggests that at least some of the older adult decrements in spatial precision occurred during encoding (Hill et al., 2020). During delayed probe trials, by which time older and younger adults had multiple experiences approaching the target from the same locations, both groups performed numerically better when approaching from a novel start point than a previously learned one. This was possibly due to interference with maintaining specific memories for a route previously taken (as all subjects were disoriented before each trial), although older adults continued to show reduced precision in their memory for the target location. Interestingly, older adults appeared to show a slight numerical advantage at novel start points compared to repeated start points (although not statistically significant), suggesting that their ability to generalize their knowledge of the hidden target outweighed their specific memories for a viewpoint. Finally, when we moved one of the distal mountain cues to determine whether participants were using a response (navigate to a single beacon) vs. allocentric strategy (navigating using multiple distal cues), we found that the groups did not differ, supporting the hypothesis that they used a mixed strategy involving both beacons and allocentric cues. We explore this issue in more detail later but attribute the relatively intact performance on allocentric trials in older adults to the emphasis on using distal cues and free ambulation in our paradigms.

In our study, we used a virtual analog of the Morris water maze, as has been used in previous studies investigating age-related differences in navigation (Moffat and Resnick, 2002; Rodgers et al., 2012; Zhong et al., 2017). A critical difference from some of these previous studies using this task in older adults is that participants had the full range of body-based cues (vestibular, somatosensory, and proprioceptive) available to them compared to desktop VR. Another important difference is that we employed large visually salient mountains as the only visible cues in the environment and would thus be something that older adults could readily detect and utilize to remember the location of the target. Finally, on every trial, we disoriented the participant by walking them around with no visual input from the head mounted display, ensuring that knowledge about their bearing did not interfere with their memory for viewpoints. We believe that these distinct methodological differences are important in explaining the apparent lack of allocentric strategy age-related impairments in our study compared to some past studies that used other versions of the Morris water maze with older adults in desktop VR.

Our findings showed that while older adults demonstrated reduced memory for the hidden location, this was true for both trajectories from start points they learned during acquisition, as well as from novel start points. These findings support the hypothesis that their representation for the hidden location, which they likely used as their basis for the memory guiding their searches, was less precise overall than younger adults. Notably, studies in patients with focal lesions to the medial temporal lobe have also noted similar deficits in representational precision. Specifically, Kolarik et al. (2016, 2018) recently demonstrated that patients with focal lesions to their hippocampus searched less precisely overall in a desktop version of the same task when compared to age-matched controls. While there are certainly several brain regions along with the hippocampus (e.g., caudate, cerebellum, prefrontal cortex, entorhinal cortex, other association cortices, and white matter tracts) that show atrophy with aging (Ge et al., 2002; Raz et al., 2005), and the virtual Morris water maze likely necessitates interactions between multiple structures (Ekstrom et al., 2017), it is interesting to note the similarities between our two studies in terms of reduced memory precision. It seems likely that gray and white matter loss that occurs with aging might underlie some of the loss in spatial precision in the older compared to younger adults (Ekstrom and Yonelinas, 2020).

During acquisition trials, when tested on repeated start locations compared to new start locations, both younger and older adults showed reduced precision in finding the hidden target. This suggests a cost in switching one’s perspective to a new viewpoint, consistent with findings from numerous other papers that suggest reduced accuracy from completely new viewpoints (Simons and Wang, 1998; May, 2004). Notably, though, and consistent with findings involving switched static viewpoints (Watanabe and Takamatsu, 2014), we did not find an age-by-viewpoint interaction effect. In other words, compared to remembering the hidden target from the same location trained during acquisition, older adults did not show a differential impairment compared to younger adults. Even when testing the reliance on multiple vs. a single distal cue, we did not find a difference between younger and older adults, suggesting both groups used beaconing and allocentric strategies to comparable extents when a single mountain moved. As mentioned previously, past VR studies arguing for a selective deficit in allocentric navigation related to aging have often employed a control task involving a response strategy of finding a proximal cue and taking an action (e.g., turning). Such strategies, though, do not necessitate representations of the location of the target and instead a stimulus-response association. Therefore, the deficits attributed to allocentric navigation reported in previous studies may have originated from reduced memory precision for the targets rather than a selective impairment in allocentric navigation (Ekstrom et al., 2014; Wolbers and Wiener, 2014).

Could it be that the lack of difference we found for repeated vs. new start points related in some form to the insufficient exposure to the repeated location or how we averaged the data? We think that this is unlikely, given our experimental design. During acquisition blocks, all participants received extensive exposure to the target (16 trials) from four repeated start locations. They then experienced a single probe trial from a different location. Here, we found that both younger and older adults showed worse performance for the novel start location, although this did not differ as a function of age. We then re-tested the same vs. different start location after a delay but this time used a completely different location than what was tested originally during acquisition or the first “acquisition probe.” Both same and different trials involved averaging across two different delayed probe trials that occurred during temporally proximate trials. Here, we found that same and different start points did not differ for either group, which was likely an effect of participants having sufficient experience with the environment that they could generalize, to some extent, the location of a target to different start points. Because participants were disoriented every trial, they had to rely on only visual information to remember a hidden target if they were to use an egocentric visual snapshot, with such egocentric memories likely to fade relatively quickly (Waller and Hodgson, 2006). Thus, we think the most likely explanation for the comparable performance on delayed egocentric and allocentric probe trials was the fading of egocentric “snapshot” memory, coupled with greater experience with the environment, allowing greater generalization. These interpretations remain exploratory, however, and future experiments will be needed to test such ideas more directly.

It is also intriguing to consider the impairments we observed here in spatial memory from the lens of episodic memory. There is wide-spread agreement that episodic memory (our memory for specific events) tends to decline with age (for a review, please see: Glisky, 2007; Cansino, 2009; Park and Reuter-Lorenz, 2009). As discussed elsewhere (Moscovitch et al., 2005), episodic and egocentric memory for specific routes likely share commonalities: they both involve remembering details specific to a single experience and involve remembering multiple sensory and visual cues. Such findings are also consistent with arguments about de-differentiation in older adults (Koen and Rugg, 2019), which refers to their difficulty with effectively differentiating the fine details of new learned information compared to young adults. It is intriguing to consider that impairments in memory for individual routes may have resulted in some of the loss of memory for starting from the same start location (egocentric) but also the ability to generalize this as effectively as younger adults to new start locations (allocentric). This is consistent with our findings suggesting that older adults showed broad impairments in spatial precision that were not selective to either egocentric or allocentric navigation. As spatial memory is important broadly to episodic memory (Robin et al., 2016), another intriguing idea is that decrements in spatial memory may underlie, in part, some of the impairments observed in older adult episodic memory. Our study did not allow us to determine the directionality of such a relationship and this issue will need to be tested in future experiments.

One previous study by Newman and Kaszniak (2000) investigated older and younger adults in a real-world version of the Morris water maze involving a 7 m tent and pole placed relative to other objects within the tent. Newman and Kaszniak reported deficits in navigation related to employing distal cues. Interestingly, older and younger adults did not differ in the initial “practice” session of remembering the pole relative to the distal objects although they did show worse performance when the distal cues were swapped. Because older adults may sometimes be more susceptible to interference than younger adults (Hasher et al., 1991), it is possible that the rearrangement of the distal cues resulted in disproportionate difficulty for the older adults. Also, because the study used the same start location for all testing, it is difficult to know whether older adults were impaired at egocentric navigation or simply having difficulty with interference. Furthermore, one study suggested intact perspective switching in older compared to younger adults (Watanabe and Takamatsu, 2014), and because perspective switching is likely one component of allocentric navigation, it seems possible that the ability to execute such an allocentric search strategy may be largely intact in older adults.

What brain mechanisms might underlie the decrements in spatial precision that we observed in older adults yet, at the same time, support intact egocentric and allocentric strategy use? Because our study was behavioral and did not involve any neural assays, we can only speculate on this issue in the hopes that such a discussion could be a useful avenue for future research. As discussed in Ekstrom and Yonelinas (2020), precision, somewhat unlike other brain processes like associative binding, might be something we expect to be distributed broadly across brain networks. This is because deficits in precision often manifest in perception, language, working memory, and other domains (Ekstrom and Yonelinas, 2020), which suggest a possible basis in network-related impairments rather than deficits attributable to a single brain region. Aging results in gray matter loss across numerous brain regions (Ge et al., 2002; Raz et al., 2005) as well as degradation of white matter tracts (Kennedy and Raz, 2009; Sullivan et al., 2010), all of which contribute to age-related impairments in cognitive function. We therefore speculate that broader changes in gray-matter and white-matter tracts could contribute to the declines in precision observed here.

In contrast, spatial strategy use involving how to use local vs. distal cues (e.g., egocentric vs. allocentric) is likely something acquired early in life and used continuously as we age involving. Decline in brain integrity with age would be less likely to affect such strategy use as this is something more likely to be dependent on well-established brain circuits and require less use of plasticity in the same way as precision might. Indeed, reports suggest that so-called “crystalized intelligence” remains remarkably stable with age, suggesting that brain circuits underlying reasoning about space could also remain largely intact (Crawford and Stankov, 1996).

In conclusion, our findings provide a potentially new perspective on navigation-related impairments in older adults. While older adults show impairments across the board in the precision of their memory for spatial locations, except when the target itself is continuously visible, our study suggests no selective deficits exist in the strategies that older adults use to remember hidden targets. In fact, our findings suggest that, given sufficient learning of a hidden target from different start points, older adults can readily generalize the location of said target from a novel start point. Our findings thus suggest a potential novel focus for navigational studies on representational precision rather than navigational strategy.

### Research Limitations

Because the sample size in our study was modest, it is difficult to speculate on a null finding between younger and older adults involving spatial strategy, and in this way, our findings remain exploratory. One possibility is that so called “super agers,” those who maintain superior memories into their 80 s, may show little deficit in spatial strategy while other healthy adults may actually show declines (Burke et al., 2019). This would best be captured by a larger sample that includes a wider range of older adults with varying spatial navigation abilities. With a larger sample, we may also find that a subset of older adults show intact spatial precision compared to younger adults. While we think that our results provide an important caveat to the idea that age-related changes impact spatial strategy and can help generate novel hypotheses about age-related changes in navigation, more work is needed to better characterize what remains intact compared to what changes with navigation and age.

## Data Availability Statement

The raw data supporting the conclusions of this article will be made available by the authors, without undue reservation.

## Ethics Statement

The studies involving human participants were reviewed and approved by University of Arizona Institutional Review Board. The patients/participants provided their written informed consent to participate in this study.

## Author Contributions

AM, AO, and AE contributed to conception and design of the study. AO, SD, and MG contributed to the recruitment and screening of older adult participants. AM organized the database. AM and YD performed the statistical analysis. AM and AE wrote the first draft of the manuscript. AM, YD, and AE wrote sections of the manuscript. All authors contributed to the manuscript revision, read, and approved the submitted version.

## Conflict of Interest

The authors declare that the research was conducted in the absence of any commercial or financial relationships that could be construed as a potential conflict of interest.
